# *Tropheryma whipplei*, *Helicobacter pylori*, and Intestinal Protozoal Co-Infections in Italian and Immigrant Populations: A Cross-Sectional Study

**DOI:** 10.3390/microorganisms10040769

**Published:** 2022-04-02

**Authors:** Lucia Moro, Elena Pomari, Martina Leonardi, Giulia La Marca, Barbara Pajola, Cristina Mazzi, Chiara Piubelli, Anna Beltrame

**Affiliations:** Department of Infectious, Tropical Diseases and Microbiology, IRCCS Sacro Cuore Don Calabria Hospital, Negrar di Valpolicella, 37024 Verona, Italy; lucia.moro@sacrocuore.it (L.M.); martina.leonardi@sacrocuore.it (M.L.); giulia.lamarca@sacrocuore.it (G.L.M.); barbara.pajola@sacrocuore.it (B.P.); cristina.mazzi@sacrocuore.it (C.M.); chiara.piubelli@sacrocuore.it (C.P.); anna.beltrame@sacrocuore.it (A.B.)

**Keywords:** *Thopheryma whipplei*, *Helicobacter pylori*, intestinal protozoa, co-infection, PCR

## Abstract

*Tropheryma whipplei* (TW), *Helicobacter pylori* (HP), and intestinal protozoa (IP) are widespread pathogens with similar routes of transmission and epidemiological risk factors. Epidemiological data on co-infection between TW, HP, and IP are scarce. We aim to more deeply investigate the co-infection rate for these pathogens, evaluating the risk factors and symptoms. Methods: This is a cross-sectional study conducted at the IRCCS Sacro Cuore Don Calabria Hospital in Northern Italy, a referral center for tropical and Whipple’s disease (WD). Stored stool samples from 143 subjects previously tested for TW DNA by real-time PCR were explored for HP and IP DNA detection. The virulence factor *cagA* was investigated in HP-positive patients. Results: A history of migration was reported significantly more in TW-positive than in negative subjects (34.1% vs. 9.1%, *p* = 0.001) and in HP-infected than in those non-infected (59.1% vs. 9.1%, *p* < 0.001). The HP infection rate differed significantly between TW-infected and uninfected groups (31.8% vs. 8.1%, *p* = 0.001), while no difference was observed for IP infection. Significantly higher TW intestinal colonization was found in HP-infected patients than in non-infected (63.6% vs. 24.8%, *p* < 0.001). In addition, the proportion of Blastocysts positive finding was also significantly higher in HP-infected than in non-infected (40.9% vs. 17.4%, *p* = 0.018). Conclusions: The present study is the first to report a high TW and HP co-infection rate. To reduce the risk of morbidity from a chronic infection of either pathogen, clinicians may consider TW-HP molecular screening on the same stool sample for patients with suspected HP disease or WD, particularly in case of travel history.

## 1. Background

*Tropheryma whipplei* (TW) is a bacterium of the Phylum *Actinobacteria* and the order Actinomycetales that causes Whipple’s disease (WD) [1]. WD is a chronic and potentially fatal illness often characterized by weight loss, diarrhea, and arthralgia, but also acute and self-limiting infections, such as acute gastroenteritis and pneumonia [2]. While the incidence of WD is very low, TW is a widespread bacterium that can colonize the gastro-intestinal tract of asymptomatic carriers [3]. TW carriage prevalence depends on the age, geographical area of residency, working activity, hygiene, and living conditions of the patients [4,5,6,7,8]. In Europe, the TW intestinal colonization rate in healthy asymptomatic adults ranges from 2 to 4% in France [6,9] and to 20% in Spain [7]. In a recent study conducted in Italy, the overall TW intestinal colonization prevalence was 6.9%, with higher rates found in the younger age group compared to that in the older (12.7 vs. 5.9%, *p* = 0.002). The prevalence was also higher in migrants from low- and middle-income countries (LMICs) than in Italians (9.3 vs. 4.9%, *p* = 0.003) [4]. This is consistent with the higher TW colonization rates reported in LMICs, especially in pediatric populations [10,11,12]. In Gabon, the overall colonization prevalence was 19.6%, declining from 40% among the 0–4 age group to 9.7% in those older than 20 years [10]. Other studies conducted in Senegal [11] and Laos [12] have confirmed these rates. This epidemiological trend, along with the fact that the relatives of WD patients have a higher prevalence of colonization [13] and the fact that humans are the only known host of TW, all support the hypothesis of human-to-human transmission [2]. Furthermore, the higher identification of TW DNA reported in Europe among sewer workers (12%) [6] and the homeless (13%) [8] suggests that transmission may be facilitated by poor sanitation.

*Helicobacter pylori* (HP) is a Gram-negative, ubiquitous bacterium that causes a common, usually lifelong infection. Although the majority of infected patients remain asymptomatic, HP infection is a major cause of peptic ulcers and non-ulcer dyspepsia and is strictly associated with gastric cancer [14]. The main pathological feature of HP derives from the presence of the cytotoxin-associated gene A (*cagA*) that is injected into host cells and is able to interact with the host cellular pathways, influencing some cellular features such as the disruption of the adherens junctions. Moreover, *cagA* is associated with gastric carcinoma [15]. HP infection prevalence is strongly correlated with socioeconomic conditions and varies greatly among countries: it ranks the highest in Africa (79.1%), then Latin America and the Caribbean (63.4%), Asia (54.7%), and finally, the lowest in North America (37.1%) and Oceania (24.4%) [16]. In Europe, the highest infection prevalence was found in the east and south (up to 84% in Poland and Portugal), while lowest in the north (18–25% in Sweden) [17]. In a recent study conducted in Italy evaluating 93 adults, of whom over 90% were migrants, 66% had an HP infection (*cagA* positives in 51% of the cases) [18]. Despite the large distribution, it remains unclear how the infection is transmitted. Nevertheless, the fecal-oral and oro-oral remain the most probable hypotheses [19].

Intestinal protozoa are a diverse group of unicellular organisms inhabiting the intestinal tract of humans and transmitted by consumption of contaminated water and food. Several species of intestinal protozoa contribute significantly to the large caseload of diarrheal diseases in humans with some causing severe debilitating illness, especially in immunosuppressed populations [20]. Higher circulations of *Entamoeba histolytica* (EH), *Entamoeba dispar* (ED), *Cryptosporidium spp*. (C), *Dientamoeba fragilis* (DF), *Giardia duodenalis* (GD), and *Blastocystis spp.* (B) are reported in LMICs [21]. In particular, intestinal protozoal infection prevalence is significantly associated with poor sanitation and water conditions [21]. The human fecal-oral transmission of these protozoa is facilitated by the large number of asymptomatic carriers that act as reservoirs.

Since TW, HP, and intestinal protozoa are likely to be transmitted in the same way and share similar risk factors, co-infections become a reasonable possibility. The results of some studies support this hypothesis [2,4,18]. A study conducted in Marseille demonstrated that 36% of the children with gastroenteritis had a TW intestinal colonization associated with other pathogens that caused the diarrhea, including GD [22]. This was confirmed in our previous study, where an infection caused by GD and/or EH was present in 72.9% of the subjects colonized by TW [4]. In a more recent study conducted in our hospital, 74% of the patients with an HP infection in an adult population composed mainly of migrants had a concomitant protozoal co-infection [18]. B and *Entamoeba coli* (EC) were the most frequent [18]. To our knowledge, no epidemiological data are available on co-infection between TW and HP.

In this study, we describe the demographic characteristics, the risk factors, the co-infections, and the symptoms in a population of patients according to the presence of TW, HP, and protozoa infections, detected by real-time PCR (rt-PCR).

## 2. Materials and Methods

### 2.1. Setting and Participants

This observational, cross-sectional study was conducted at the Department of Infectious, Tropical Diseases and Microbiology (DITM) of the IRCCS Sacro Cuore Don Calabria Hospital in Negrar di Valpolicella (Verona), northeastern Italy. The DITM laboratory is a referral center for tropical and parasitic infections that analyses stool specimens from a large variety of patients, including those arriving or returning from all over the world (i.e., migrants and travelers). Since 2015, the DITM laboratory has also become a reference center for rare diseases such as WD.

Stools from adults previously tested for TW by molecular analysis were used for the study. These specimens were collected from July 2014 to February 2021 and stored at −80 °C in the BioBank (Tropica Biobank, BBMRI-eric ID: IT_1605519998080235) for possible future investigations. Subjects’ inclusion criteria for TW DNA analyses were: patients with symptoms suggestive of WD (*n* = 106, 74.1%); asymptomatic patients with a TW intestinal colonization identified in our previous study [4] and requiring a laboratory follow-up (*n* = 32, 22.4%); cohabiting family members of WD patients who required a screening (*n* = 3, 2.1%); and patients with a diagnosis of WD (*n* = 2, 1.4%). The stored samples were tested afterwards for DNA detection of HP, EH, ED, C, DF, GD, and B. No microscopy data about intestinal protozoa were available. Stool samples positive for HP DNA were also analyzed for *cagA* virulence factor.

Demographic characteristics (age, sex), risk factors (geographic origin and travel in LMICs for more than one month), and symptom data were retrieved from the electronic database of the hospital.

### 2.2. Laboratory Methods

#### 2.2.1. DNA Isolation

Total DNA was extracted from 250 mg of stool using the MagNA Pure LC 2.0 Instrument (Roche, Monza, Italy). In each sample, Phocine Herpes Virus type-1 (PhHV-1) was added as internal control for the isolation and amplification steps, as described previously [4,23].

#### 2.2.2. Real-Time PCR Analysis

All the amplification reactions were performed using 5 µL of DNA and using the SsoAdvanced universal probes supermix (BioRad, Milan, Italy). All reactions and data analyses were performed on the CFX96 system (BioRad, Milan, Italy).

For TW, detection was performed using a first rt-PCR assay (with the primer pair TW27-F and TW182-R, and probe 27F-182R), targeting a 105 bp repeated sequence of the bacterium. If the result of this first assay was positive, it was systematically confirmed by a second rt-PCR assay (with the primer pair TW13-F and TW163-R, and probe 13F-163R) targeting a different DNA sequence. The primers/probe sets for TW are reported in Supplemental Table S1. For both rt-PCR assays, the program consisted of an initial step of 3 min at 95 °C, followed by 40 cycles of 15 s at 95 °C, and 60 s at 60 °C [4,6,22].

For HP, the rt-PCRs were performed for targeting *ureC* (*glmM*) and *cagA.* The primers/probe sets for HP are reported in Supplemental Table S2. The program consisted of an initial step of 3 min at 95 °C, followed by 50 cycles of 15 s at 95 °C, 30 s at 58 °C, and 30 s at 72 °C [24].

Moreover, according to the routine procedure of our laboratory, molecular diagnostic screening for intestinal protozoa was performed by two separate multiplex rt-PCRs for EH—ED—C, and for GD—DF—B. Multiplex rt-PCRs were performed by adapting the reported protocols [25,26,27,28,29]. The primers/probe sets are reported in Supplemental Table S3. The program consisted of an initial step of 3 min at 95 °C, followed by 40 cycles of 15 s at 95 °C, 30 s at 60 °C, and 30 s at 72 °C.

#### 2.2.3. Statistical Analysis

Descriptive statistics were used to describe the overall population (*n* = 143) and to describe patients stratified according to the results of the rt-PCR for TW, HP, and protozoal infections. Categorical variables were reported as absolute and relative frequencies. Age was summarized as median and interquartile range [IQR]. Chi-square with the Monte Carlo simulated *p*-value test were used to compare the proportions between the presence of infections and demographic and clinical variables. All estimations were reported with 95% confidence intervals. The statistical level of significance was set at 5%. Data analysis was performed with SAS software version 9.4 (Cary, NC, USA).

## 3. Results

We analyzed 143 stool samples. The demographic characteristics and the risk factors considered are shown in Table 1.

The median age was 48 years (IQR 36, 62); 80 (55.9%) were males, and 24 (16.8%) were immigrants. In the overall population, a history of travel in LMICs was reported by 35 (24.5%) patients. Most of the migrants came from Africa (75%), which was also the continent most visited by travelers (62.9%).

Sixty-seven (46.9%) stool samples resulted negative for all pathogens. On the other hand, 44 (30.8%) were rt-PCR-positive for TW, 22 (15.4%) for HP, and 43 (30%) for at least one intestinal protozoan (8 *GD*, 30 B, 8 DF, 6 ED), as shown in Table 1. C and EH were not detected. Fourteen out of 22 (63.6%) patients with a positive rt-PCR HP tested positive for *cagA*+.

Immigrant patients had higher rates of TW and HP infections compared to those in Italians (62.5% vs. 24.3%, *p* < 0.001 and 54.1 vs. 7.6%, *p* < 0.001, respectively). The co-infection with two or at least three pathogens was found in 30 (21%) and 8 (5.6%) patients, respectively. Seven patients (4.9%) presented a co-infection of TW, HP, and at least one parasite. Among them, 100% carried only B, 28.5% carried B and GD, 28.5% B and ED, 14.2% B, GD, and ED.

Symptoms were reported by 114 (79.7%) patients (Table 1). This information was not available for 17 patients (11.9%). The combination of gastrointestinal symptoms, arthralgia, fever, and weight loss was reported in one patient (0.7%), while the combination of gastrointestinal symptoms, arthralgia, and weight loss was reported in eight patients (5.6%).

The characteristics of the patients according to the results of the rt-PCR for TW are shown in Table 2.

A history of migration was significantly more reported in TW-positive than in negative patients (34.1% vs. 9.1%, *p* = 0.001). HP infection rate differed significantly between TW-infected and uninfected patients (31.8% vs. 8.1%, *p* = 0.001). No difference was found in the case of intestinal protozoa infection except for GD, although the GD difference was not statistically significant. While the symptoms were most common overall in patients not infected by TW, weight loss was more reported in TW-positive versus negative patients (40.9% vs. 24.2%, *p* = 0.048).

The characteristics of the patients according to the results of the rt-PCR for HP are shown in Table 3. Younger age and male sex were reported more in patients with an HP infection than in those without (49 vs. 35 years, *p* = 0.044 and 81.8% vs. 51.2%, *p* = 0.010, respectively).

In addition, a history of migration was more reported in patients with an HP infection than in those without (59.1% vs. 9.1%, *p* < 0.001). Significantly higher TW intestinal colonization was found in patients with an HP infection than in those without (63.6% vs. 24.8%, *p* < 0.001). The B infection rate was significantly higher in HP-positive than in negative patients (40.9% vs. 17.4%, *p* = 0.018). No symptoms were reported with more frequency in patients with an HP infection. *(p* = 0.001).

Among patients presenting TW, HP, and at least one parasite co-infection, all reported history of migration or travel in LMICs, and three out of seven (42.9%) had symptoms.

No significant differences were found between patients according to the results of rt-PCR for at least one protozoan (Table 4).

## 4. Discussion

This observational study is, to our knowledge, the first to investigate co-infections between TW, HP, and intestinal protozoa. These are all pathogens with possible fecal-oral and/or oro-oral transmission.

Our population study included adults with TW intestinal colonization, WD, or with a clinical suspicion of WD. Most patients were Italian, and less than a quarter reported travel in tropical areas or LMICs for more than one month. Nearly 80% of the patients had symptoms, most being gastrointestinal. Overall, 15.4% and 5.6% of the patients had a co-infection with at least two or three pathogens, respectively, and 4.9% of patients carried TW, HP, and at least one parasite co-infection at the same time.

A history of migration was significantly more reported by patients with TW intestinal colonization or HP infection. This was expected, considering that these pathogens are more frequent in emigrant countries and that their prevalence is known to be highly dependent on socioeconomic conditions. Most patients had emigrated from Africa or had traveled to Africa for vacation. The majority of the available epidemiological data on TW infection comes from studies conducted in Africa, predominantly in younger age groups [10,11]. In Senegal, the prevalence of TW colonization is 17.4% among subjects older than 10 years [30], whereas in Gabon, it is 9.7% among subjects over 20 years of age [10]. The highest prevalence of HP infection worldwide is also found in Africa (87.7% in Nigeria) [16]. In our study, no difference in migration history was found in patients with or without protozoal infection, probable due to the small study sample, since few patients resulted positive for protozoa.

Patients with TW intestinal colonization or WD were co-infected with HP in 31.8% of cases and were mainly carriers of *cagA* (22.7%). Although these pathogens have similar epidemiological risk factors, as well as the same probable mode of transmission, this is the first time that this co-infection has been reported to our knowledge. In fact, the mode of HP transmission is still unclear [19], although fecal-, oro-, or gastro-oral are the most likely routes. These same transmission pathways are believed to be more probable for TW [31,32]. As it may cause chronic infection, HP is an important risk factor for duodenal and gastric ulcers, mucosal atrophy, gastric carcinoma, and gastric lymphoma, especially if the HP carries *cagA* [33].

It would be interesting to understand the role of co-infection in the natural history of HP infections. Since an early diagnosis and treatment of chronic HP infection can prevent significant morbidity and mortality [34,35], endoscopy with gastric biopsies is recommended in the presence of upper gastrointestinal symptoms, particularly unintentional weight loss or gastrointestinal bleeding [14]. Although WD is a rare disease, physicians should be aware of the likelihood of finding HP co-infection in these patients.

Thirty-six percent of patients with TW DNA in their stool samples were also co-infected with at least one intestinal protozoan. This was particularly evident for GD. The strong relationship between these two pathogens has already been described by other authors [4,22,36]. In a previous study, we found that GD and EH were risk factors for TW infection (OR 3.60; 95% CI 1.79–6.92 and OR 8.65 95% CI 4.18–17.46, respectively) [4]. Considering that weight loss was significantly more reported in patients colonized by TW or with WD, screening for GD in these patients becomes advisable. In fact, GD itself can be a cause of malabsorption, with detrimental nutritional effects [37].

Sixty-four percent and 45% of patients with HP DNA in their stools were co-infected with TW and at least one intestinal protozoan, respectively. A chronic TW infection may evolve into WD, presenting as joint symptoms, diarrhea (75%), malabsorption, and weight loss (80–90%) [2]. If a clinical diagnosis of WD is suspected, multiple biopsies in different areas of the duodenum are recommended [2]. The molecular test to detect HP and TW DNA in a stool sample is an advantageous, non-invasive test that allows the physician to select patients who must undergo endoscopy with gastric and/or duodenal biopsies. In addition to having excellent sensitivity and specificity for the diagnosis of both pathogens [24,38], a rt-PCR in the stool sample is able to detect specific mutations for antibiotic resistance for HP and HP virulence factor [39].

Co-infection between HP and intestinal parasites has been described previously [18,40,41]. In our study, B was present in 40.9% of the patients infected by HP, and 17.4% among those HP-negative. This prevalence was lower than the prevalence reported in our previous study (67%) [18], probably due to the diverse study population (fewer immigrants in the current study). The co-infections observed between patients with TW or HP infections and intestinal protozoa could be a consequence of the “carrier” role of protozoa, which has already been described for amoeba with *Legionella pneumophila* [42]. This may serve as another experimental hypothesis.

The present study has several limitations. The first is its retrospective setting, which did not allow for a definitive conclusion. Second, our cohort was small and quite heterogeneous, with different ages, origins, and clinical characteristics. Third, data on viral infections were not available, as well as complete information was missing for some patients. Finally, the samples analyzed here were selected from specific patient types, which prevents generalizing the results to the general population. Nevertheless, our results can provide the basis for further analyses and for studies assessing the potential role of helminth co-infections in this study population.

## 5. Conclusions

To our knowledge, the present study is the first to report a high TW and HP co-infection rate. To reduce the risk of morbidity from a chronic infection of both pathogens, clinicians may propose TW-HP screening with molecular testing on the same stool sample for all patients with suspected HP disease or WD, particularly if a history of travel is reported. Larger prospective studies are needed to better illuminate the prevalence of co-infection, as well as the validity of using molecular biology in stool samples to diagnose both infections early.

## Figures and Tables

**Table 1:** Demographic data, risk factors, rate of infections (TW, HP, and protozoa), and symptoms of the overall population (*n* = 143). TW, *T. whipplei*; HP, *H. pylori*; GD, *G. duodenalis*; B, *Blastocystis spp.*; C, *Cryptosporidium spp.*; DF, *D. fragilis*; ED, *E. dispar*; EH, *E. histolytica*; LMICs, low- and middle-income countries; IQR, interquartile range.

	Overall
Age, years, median [IQR]	48 [36–62]
Male, *n/*N (%)	80/143 (55.9)
Travel in tropical or LMICs, *n/*N (%)	35/143 (24.5)
Africa *n/*N (%)	22/35 (62.9)
America *n/*N (%)	13/35 (37.1)
Asia *n/*N (%)	15/35 (42.9)
Others *n/*N (%)	3/35 (8.6)
Migrants, *n/*N (%)	24/143 (16.8)
From Africa *n/*N (%)	18/24 (75.0)
From America *n/*N (%)	2/24 (8.3)
From Asia *n/*N (%)	1/24 (4.2)
From Eastern Europe *n/*N (%)	2/24 (8.3)
rt-PCR TW, positive, *n/*N (%)	44/143 (30.8)
rt-PCR HP, positive, *n/*N (%)	22/143 (15.4)
rt-PCR HP *cagA,* positive, *n/*N (%)	14/143 (9.8)
rt-PCR for at least one intestinal protozoa, positive, *n/*N (%)	43/143 (30.1)
GD *n/*N (%)	8/143 (5.6)
B *n/*N (%)	30/143 (21.0)
C *n/*N (%)	0/143 (0)
DF *n/*N (%)	8/143 (5.6)
ED *n/*N (%)	6/143 (4.2)
EH *n/*N (%)	0/143 (0)
Co-infection with two or more pathogens, *n/*N (%)	30/143 (21)
Co-infection with three or more pathogens, *n/*N (%)	8/143 (5.6)
Symptoms, *n/*N (%)	114/143 (79.7)
Gastrointestinal symptoms, *n/*N (%)	76/143 (53.1)
Arthralgia, *n/*N (%)	35/143 (24.5)
Fever, *n/*N (%)	35/143 (24.5)
Weight loss, *n/*N (%)	42/143 (29.4)
Other, *n/*N (%)	58/143 (40.6)

Continuous variables are expressed as median (IQR). Categorical variables as *n*/N (%). Where values do not add up to 100%, the remaining data are missing.

**Table 2:** Demographic data, risk factors, rate of infections (HP and protozoa), and symptoms of 143 patients, according to the results of the rt-PCR for TW. TW, *T. whipplei*; HP, *H. pylori*; GD, *G. duodenalis*; B, *Blastocystis spp.*; C, *Cryptosporidium spp.*; DF, *D. fragilis*; ED, *E. dispar*; EH, *E. histolytica*; LMICs, low- and middle-income countries; IQR, interquartile range.

	rt-PCR TW Negative N = 99	rt-PCR TW Positive N = 44	*p* Value
Age, years, median [IQR]	49 [37–63]	44 [35–60]	*p* = 0.329 ^†^
Male, *n* (%)	50 (50.5)	30 (68.2)	*p* = 0.063 ^§^
Travel in tropical or LMICs, *n* (%)	22 (22.2)	13 (29.5)	*p* = 0.292 ^§^
Migrants, *n* (%)	9 (9.1)	15 (34.1)	*p* = 0.001 ^§^
rt-PCR HP, positive, *n* (%)	8 (8.1)	14 (31.8)	*p* = 0.001 ^§^
rt-PCR HP *cagA,* positive, *n* (%)	4 (5.0)	10 (22.7)	*p* = 0.402
rt-PCR for at least one intestinal protozoan, positive, *n* (%)	27 (27.3)	16 (36.4)	*p* = 0.325 ^§^
GD, *n* (%)	3 (3.0)	5 (11.4)	*p* = 0.052 ^§^
B, *n* (%)	18 (18.2)	12 (27.3)	*p* = 0.283 ^§^
C, *n* (%)	0 (0)	0 (0.0)	
DF, *n* (%)	7 (7.1)	1 (2.3)	*p* = 0.426 ^§^
ED, *n* (%)	3 (3.0)	3 (6.8)	*p* = 0.382 ^§^
EH, *n* (%)	0 (0.0)	0 (0)	
Symptoms, *(n*%)	83 (83.8)	31 (70.5)	*p* < 0.001 ^§^
Gastrointestinal symptoms, *n* (%)	58 (58.6)	18 (40.9)	*p* = 0.010 ^§^
Arthralgia, *n* (%)	26 (26.3)	9 (20.5)	*p* = 0.534 ^§^
Fever, Yes, *n* (%)	24 (24.2)	11 (25.0)	*p* = 1.000 ^§^
Weight loss, *n* (%)	24 (24.2)	18 (40.9)	*p* = 0.048 ^§^
Other, *n* (%)	42 (42.4)	16 (36.4)	*p* = 0.580 ^§^

N is the number of non-missing values. ^†^ Wilcoxon–Mann–Whitney test. ^§^ Chi-square test. Continuous variables are expressed as: median [IQR]; categorical variables as *n* (%).

**Table 3:** Demographic data, risk factors, rate of infections (TW and protozoa), and symptoms of 143 patients, according to the results of the rt-PCR for HP. TW, *T. whipplei*; HP, *H. pylori*; GD, *G. duodenalis*; B, *Blastocystis spp.*; C, *Cryptosporidium spp.*; DF, *D. fragilis*; ED, *E. dispar*; EH, *E. histolytica*; LMICs, low- and middle-income countries; IQR, interquartile range.

	rt-PCR HP Negative N = 121	rt-PCR HP Positive N = 22	*p* Value
Age, years, median, [IQR]	49 [38–62]	35 [28–55]	*p* = 0.044 ^†^
Male, *n* (%)	62 (51.2)	18 (81.8)	*p* = 0.010 ^§^
Travel in tropical or LMICs	33 (27.3)	2 (9.1)	*p* = 0.165 ^§^
History of migration, *n* (%)	11 (9.1)	13 (59.1)	*p* < 0.001 ^§^
rt-PCR TW positive, *n* (%)	30 (24.8)	14 (63.6)	*p* < 0.001 ^§^
rt-PCR for at least one intestinal protozoa, positive, *n* (%)	33 (27.3)	10 (45.5)	*p* = 0.118 ^§^
GD, *n* (%)	5 (4.1)	3 (13.6)	*p* = 0.106 ^§^
B, *n* (%)	21 (17.4)	9 (40.9)	*p* = 0.018 ^§^
C, *n* (%)	0 (0)	(0.0)	
DF, *n* (%)	8 (6.6)	0 (0.0)	*p* = 0.352 ^§^
ED, *n* (%)	4 (3.3)	2 (9.1)	*p* = 0.233 ^§^
EH, *n* (%)	0 (0)	0 (0)	
Symptoms, *n* (%)	102 (84.3)	12 (54.5)	*p* = 0.001 ^§^
Gastrointestinal symptoms, *n* (%)	70 (57.9)	6 (27.3)	*p* = 0.024 ^§^
Arthralgia, *n* (%)	31 (25.6)	4 (18.2)	*p* = 0.597 ^§^
Fever, *n* (%)	33 (27.3)	2 (9.1)	*p* = 0.090 ^§^
Weight loss, *n* (%)	38 (31.4)	4 (18.2)	*p* = 0.309 ^§^
Other, *n* (%)	51 (42.1)	7 (31.8)	*p* = 0.494 ^§^

N is the number of non-missing values. ^†^ Wilcoxon–Mann–Whitney test. ^§^ Chi-square test. Continuous variables are expressed as median [IQR]; categorical variables as *n* (%).

**Table 4:** Demographic data, risk factors, rate of infections (TW and HP), and symptoms of 143 patients, according to the results of the rt-PCR for the detection of at least one protozoan. TW, *T. whipplei*; HP, *H. pylori*; LMICs, low- and middle-income countries; IQR, interquartile range.

	rt-PCR Protozoa Negative N = 100	rt-PCR Protozoa Positive N = 43	*p* Value
Age, years, median [IQR]	47 [36–61]	48 [34–64]	*p* = 0.810 ^†^
Male, *n* (%)	53 (53.0)	27 (62.8)	*p* = 0.332 ^§^
Travel in tropical or LMICs, *n* (%)	20 (20.0)	15 (34.9)	*p* = 0.119 ^§^
Migrants, *n* (%)	14 (14.0)	10 (23.3)	*p*= 0.213 ^§^
rt-PCR TW, positive, *n* (%)	28 (28.0)	16 (37.2)	*p* = 0.348 ^§^
rt-PCR HP, positive, *n* (%)	12 (12.0)	10 (23.3)	*p* = 0.129 ^§^
rt-PCR HP *cagA,* positive, *n* (%)	7 (7.0)	7 (16.3)	*p* = 0.685
Symptoms, *n* (%)	85 (85.0)	29 (67.4)	*p* = 0.012 ^§^
Gastrointestinal symptoms, *n* (%)	58 (58.0)	18 (41.9)	*p* = 0.208 ^§^
Arthralgia, *n* (%)	30 (30.0)	5 (11.6)	*p* = 0.021 ^§^
Fever, *n* (%)	28 (28.0)	7 (16.3)	*p* = 0.158 ^§^
Weight loss, *n* (%)	32 (32.0)	10 (23.3)	*p* = 0.327 ^§^
Other, *n* (%)	44 (44.0)	14 (32.6)	*p* = 0.250 ^§^

N is the number of non-missing values. ^†^ Wilcoxon–Mann–Whitney test. ^§^ Chi-square test. Continuous variables are expressed as median [IQR]; categorical variables as *n* (%).

## Data Availability

All data generated or analyzed during this study are included in this published article (and its Supplementary Information Files).

## Supplementary Materials

The following supporting information can be downloaded at: https://www.mdpi.com/article/10.3390/microorganisms10040769/s1, Table S1: Primers and probes used for TW rt-PCR assays. Table S2: Primers and probes used for HP rt-PCR assays. Table S3: Primer/probe sets of two multiplex rt-PCRs for intestinal protozoa.
