# Supplementary material for: Tropheryma whipplei, Helicobacter pylori, and Intestinal Protozoal Co-Infections in Italian and Immigrant Populations: A Cross-Sectional Study
Source: Microorganisms. 2022 Apr 2;10(4):769. doi: 10.3390/microorganisms10040769 (PMC9027763; doi:10.3390/microorganisms10040769)
Supplement: Supplementary file 1 [file microorganisms-10-00769-s001.zip › microorganisms-1648729-supplementary.pdf]

TableS1. Primers and probes used for TW rt-PCR assays.

	Primer/probe name	Primer/probe sequence	Ref
First rtPCR	TW27-F	5’-TGTTTTGTACTGCTTGTAACAGGATCT-3’	[4, 6]
	TW182-R	5’-TCCTGCTCTATCCCTCCTATCATC-3’	
	ROX-TW27F-182R-BHQ2	5’-AGAGATACATTTGTGTTAGTTGTTACA-3’	
Second rtPCR	TW13-F	5’-TGAGTGATGGTATGTCTGAGAGATATGT-3’	[4, 6]
	TW163-R	5’-TCCATAACAAAGACAACAACCAATC-3’	
	FAM-TW13F-163R-TAMRA	5’-AGAAGAAGATGTTACGGGTTG-3’	

Table S2. Primers and probes used for HP rt-PCR assays.

Primer/probe name	Primer/probe sequence	Ref
ureC-F	5’-TGAGCGAATGCATGCGATT-3’	
ureC-R	5’-AATGATATGCCCGCTTTGCT-3’	[24]
FAM-ureC-MGBEQ	5’-ACAAAGCCAATTTTGGAGG-3’	
cagA-F	5’-TCAAGAACCAGTTCCCCATGTC-3’	
cagA-R	5’-TCTCTAGCTTCAGGCGGTAAGC-3’	[24]
HEX-cagA-MGBEQ	5’-ACCAGATATAGCCACTACC-3’	

Table S3. Primer/probe sets of two multiplex rt-PCRs for intestinal protozoa.

Multiplex	Parasite	Primers/probe	Ref
1	*Entamoeba histolytica*	For 5'-ATTGTCGTGGCATCCTAACTCA-3'<br>Rev 5'-GCGGACGGCTCATTATAACA-3'<br>Probe 5'-FAM-TCATTGAATGAATTGGCCATTT-3'-MGB	[25]
	Entamoeba dispar	For 5'-ATTGTCGTGGCATCCTAACTCA-3'<br>Rev 5'-GCGGACGGCTCATTATAACA-3'<br>Probe 5'-HEX-TTACTTACATAAATTGGCCACTTTG-3'-MGB	[25]
	Cryptosporidium spp.	For 5'-ATGAGCGGGTAACGGGGAAT-3'<br>Rev 5'-CCAATTACAAAACCAAAAAGTCC-3'<br>Probe 5'-CY55-CGCGCCTGCTGCCTTCTTTAGATG-3'-BBQ	[26]
2	*Giardia intestinalis*	For 5'-GACGGCTCAGGACAACGGTT-3'<br>Rev 5'-TTGCCAGCGGTGTCCG-3'<br>Probe 5'-CY55-CCCGCGGCGGTCCCTGCTAG-3'-BHQ	[27]
	Dientamoeba fragilis	For 5'-CAACGGATGTCTTGGCTCTTTA-3'<br>Rev 5'-TTGCCAGCGGTGTCCG-3'<br>Probe 5'-HEX-CAATTCTAGCCGCTTAT-3'-MGB	[28]
	Blastocystis spp.	For 5'-GGTCCGGTGAACACTTTGGATTT-3'<br>Rev 5'-CCTACGGAAACCTTGTTACGACTTCA-3'<br>Probe 5'-FAM-TCGTGTAAATCTTACCATTTAGAGGA-3'-MGB	[29]
